# Geographic variation in the distribution of *Anaplasma phagocytophilum* variants in host-seeking *Ixodes scapularis* nymphs and adults in the eastern United States elucidated using next generation sequencing

**DOI:** 10.1016/j.ttbdis.2024.102360

**Published:** 2024-05-30

**Authors:** Andrias Hojgaard, Erik Foster, Sarah E. Maes, Lynn M. Osikowicz, Christina M. Parise, Joel Villalpando, Rebecca J. Eisen

**Affiliations:** Division of Vector-Borne Diseases, Centers for Disease Control and Prevention, Fort Collins, CO, USA

**Keywords:** Deer ticks, Tick-borne diseases, Tick surveillance, Anaplasma phagocytophilum

## Abstract

Human anaplasmosis cases, caused by *Anaplasma phagocytophilum*, are increasing in the United States. This trend is explained, in part, by expansion in the geographic range of the primary vector, *Ixodes scapularis*. Multiple variants of *A. phagocytophilum* have been identified in field collected ticks, but only a single variant (human active, or “Ap-ha,” variant) has been shown to be pathogenic in humans. Until recently, laboratory methods used to differentiate variants were cumbersome and seldomly used in large scale assessments of the pathogen’s geographic distribution. As a result, many surveys reported *A. phagocytophilum* without segregating variants. Lack of discrimination among *A. phagocytophilum* variants could lead to overestimation of anaplasmosis risk to humans. Next Generation Sequencing (NGS) assays were recently developed to efficiently detect multiple *Ixodes scapularis*-borne human pathogens including Ap-ha. In this study, we utilized NGS to detect and differentiate *A. phagocytophilum* variants (Ap-ha vs. non ha) in host-seeking *I. scapularis* nymphs and adults collected across 23 states in the eastern United States from 2012 to 2023 as part of national tick surveillance efforts and research studies. Many of the included ticks were tested previously using a TaqMan PCR assay that could detect *A. phagocytophilum* but could not differentiate variants. We retested *A. phagocytophilum* infected ticks with NGS to differentiate variants. *Anaplasma phagocytophilum* (any variant) was identified in 165 (35 %) of 471 counties from which ticks were tested, whereas Ap-ha was detected in 70 (15 %) of 469 counties where variants were differentiated. Both variants were identified in 32 % (*n* = 40) of 126 counties with either variant detected. Among states where *A. phagocytophilum* (any variant) was detected, prevalence ranged from 2 % to 19 % in unfed adults and from 0.2 % to 7.8 % in unfed nymphs; prevalence of Ap-ha variant ranged from 0.0 % to 16 % in adults, and 0.0 % to 4.6 % in nymphs.

## 1. Introduction

In the United States (US), pathogens spread by the blacklegged tick, *Ixodes scapularis*, account for the majority of human vector-borne disease cases reported to the US Centers for Disease Control and Prevention (CDC) (NNDSS, 2023). The incidence and distribution of *I. scapularis*-borne diseases, including Lyme disease, anaplasmosis, and babesiosis have increased since becoming notifiable conditions (Eisen and Eisen, 2018; NNDSS, 2023). This trend is partially attributable to changes in the population density and geographic distribution of *I. scapularis* and its associated pathogens. Over the past half century, *I. scapularis* has continued to reclaim its historic range covering most of the eastern US (Eisen et al., 2016; Fish, 2022; Eisen and Eisen, 2023). The distributions of human disease-causing pathogens spread by the blacklegged tick are more limited than the distribution of the tick but also appear to be expanding in range (Fleshman et al., 2022; Prusinski et al., 2023), putting an increased number of communities at risk for tick-borne diseases. Currently, *Borrelia burgdorferi* sensu stricto (s.s.), the causative agent of Lyme disease is the most prevalent and widely distributed pathogen found in *I. scapularis*, with a distribution focused primarily in northern states of the eastern US. *Anaplasma phagocytophilum* (anaplasmosis) has a similarly wide distribution but is typically detected at lower prevalence compared with *B. burgdorferi* s.s. (Porter et al. 2021; Foster et al., 2023).

Subsequent to *A. phagocytophilum* being recognized as the causative agent of human granulocytic anaplasmosis, it became clear that *I. scapularis* carried multiple variants of the bacteria, but not all were pathogenic in humans. Multiple variants have been described in *I. scapularis* (Massung et al., 1998, 2002; Courtney et al., 2003; Lee et al., 2014; Keesing et al., 2014; Liveris et al., 2021; Hojgaard et al., 2022; Prusinski et al., 2023), but only one variant (referred to as the human active variant or “Ap-ha”) has been detected from clinical samples (Massung et al., 1998; Liveris et al., 2021). Laboratory techniques used to differentiate the strains, commonly traditional PCR assays with follow up sequencing (Massung et al., 1998; Courtney et al., 2003; Lee et al., 2014), are cumbersome and consume large volumes of limited DNA. As a result, many previous assessments of the distribution and prevalence of *A. phagocytophilum* in ticks did not differentiate variants (Prusinski et al., 2014; Xu et al., 2016, 2019; Johnson et al., 2018; Lehane et al., 2021; Rounsville et al., 2021; Fleshman et al., 2022; Foster et al., 2023), or they differentiated variants but focused on limited spatial scales (Massung et al., 2002; Courtney et al. 2003; Lee et al. 2014; Keesing et al. 2014; Prusinski et al., 2023). These studies demonstrated that although in some localities variants co-occur, in others only a single variant may be detected; thus, grouping variants in risk assessments may inflate risk to humans.

The initial study that described Ap-ha and variant 1 (a non-human active variant) focused on the 16S rRNA gene of *A. phagocytophilum* (Massung et al., 2002). Recent studies described additional genetic targets that accurately differentiate the variants (Liveris et al., 2021; Hojgaard et al., 2022). One of the informative targets (*gro*EL) was incorporated into a next generation sequencing (NGS) multiplex assay designed to detect and differentiate bacterial and protozoan pathogens found in *Ixodes* species ticks (Hojgaard et al., 2020; Osikowicz et al., 2024). This innovation increased efficiency over traditional PCR and sequencing and enabled assessments of the distribution and prevalence of *I. scapularis*-borne pathogens across the US. As a component of national tick surveillance efforts, CDC tests thousands of *I. scapularis* ticks per year to support public health partners in defining when and where people are at risk for exposure to tick-borne pathogens. From 2015 through 2022, testing relied on a TaqMan testing algorithm that detected *A. phagocytophilum* but did not differentiate variants (Graham et al., 2018). Based on that testing, and testing by partners, county level maps showing the distribution and prevalence of *A. phagocytophilum* (variants not differentiated) were published recently (Fleshman et al., 2022; Foster et al., 2023). To improve characterization of risk to humans, in this study, we 1) retested host-seeking *I. scapularis* nymphs and adults that were identified by CDC as infected with *A. phagocytophilum* by TaqMan PCR using a NGS assay described here to differentiate variants, 2) tested host-seeking ticks submitted in 2023 using NGS multiplex assays to detect and differentiate *A. phagocytophilum* variants (Hojgaard et al., 2020; Osikowicz et al., in2024), and 3) combined these testing results for ticks collected from 2012 to 2023 across the eastern US to report the county-level distribution of *A. phagocytophilum* by variant and the state-level prevalence by variant and tick life stage.

## 2. Materials and methods

### 2.1. Origin of ticks and reference DNA and description of TaqMan testing

Host-seeking ticks were submitted to the CDC Division of Vector-Borne Diseases (DVBD) in Fort Collins, Colorado for tick-borne pathogen screening. Unfed *I. scapularis* nymphs and adults collected by drag or flag sampling between 2012 and 2022 were tested individually for five human pathogens (*B. burgdorferi* sensu stricto, *Borrelia mayonii, Borrelia miyamotoi, A. phagocytophilum*, and *Babesia microti*) using a TaqMan PCR testing algorithm that detected *A. phagocytophilum*, but did not differentiate variants (Graham et al., 2018). A sample was considered positive for *A. phagocytophilum* if it was positive by TaqMan PCR for two *A. phagocytophilum* specific PCR targets (p44 and msp4) and positive for the tick actin housekeeping gene (Graham et al., 2018). A sample was called negative for *A. phagocytophilum* if the tick actin target was positive and one or both of the *A. phagocytophilum* targets were negative. Samples were called inconclusive if the tick actin target was negative and the *A. phagocytophilum* targets were negative by TaqMan PCR (Graham et al., 2018). In this study, specimens that were identified as *A. phagocytophilum* positive by the TaqMan assay, and sufficient amount of sample DNA was still available to perform the new assay, were retested to differentiate *A. phagocytophilum* variants using a NGS multiplex PCR amplicon sequencing (MPAS) assay described below (Section 2.2). The tick specimens that were positive for *A. phagocytophilum* with the TaqMan assay but did not contain sufficient DNA for retesting were still included in the overall county level summaries of *A. phagocytophilum* “any variant” presence and prevalence, but they were excluded from variant-specific summaries (described in Sections 2.6 and 2.7).

The reference DNA produced from cultures of *A. phagocytophilum* strain USG3 and *A. phagocytophilum* strain V1 and used as controls in this study were provided by the Rickettsial Zoonoses Branch, Division of Vector-Borne Diseases, CDC, Atlanta, GA. The strain USG3 is representative of the Ap-ha variant known to be pathogenic in humans and strain V1 represents a variant that has not been shown to be pathogenic in humans (Ap-non ha) (Nicholson et al., 1997).

### 2.2. Retesting of A. phagocytophilum-infected I. scapularis nymphs and adults with new MPAS assay to differentiate variants

We retested *I. scapularis* DNA samples that were previously identified by CDC as infected with *A. phagocytophilum* by TaqMan PCR, using a new MPAS assay. This MPAS assay includes two PCR targets, *sdhC* and *groEL*, that have previously been shown to accurately distinguish Ap-ha and Ap-non ha variants (Hojgaard et al., 2022; Liveris et al., 2021).

The PCR reactions for the MPAS experiments were performed in 25 μl, which included 12.5 μl TEMPase 2x master mix (AMPLICON, Denmark), 5 μl tick nucleic acids extract and 7.5 μl PCR primers resuspended in PCR grade water (see Table 1 for primers and primer concentrations). Cycling conditions consisted of 95 °C for 15 min to denature DNA followed by 40 cycles of 95 °C for 20 s, 58 °C for 20 s and 72 °C for 1 min, ending with a 5 min incubation at 72 °C, using a C1000 Touch thermal cycler (BioRad, Hercules, CA). The PCR amplicon libraries from the PCR reactions were then generated as described in Osikowicz, et al. (Osikowicz et al.,2024). Briefly, following the initial PCR, samples were purified using a KingFisher Flex System (Thermo Fisher Scientific, Waltham, MA), Nextera XT Indexes (Illumina, San Diego, CA) were added, samples were purified, pooled, and the concentration was assessed with a Qubit 4 (Thermo Fisher Scientific) (Osikowicz et al.,2024). Finally, sequencing was performed on the MiSeq system (Illumina) using MiSeq Reagent Kits Nano 500V2 according to the manufacturer’s protocol (Illumina).

### 2.3. Comparison of AP-ha and AP-non ha variant classification using sdhC and groEL PCR targets

Based on a limited sample size, a previous study demonstrated consistency in variant classification based on *sdhC* and *groEL* (Hojgaard et al., 2022). Here, we further evaluated variant classification through pairwise comparisons of tick samples tested using the duplex PCR described in the previous section (Section 2.2). This was conducted to demonstrate the validity of basing variant classifications on the single target (*groEL*) that is incorporated in the multi-pathogen detection assay used for routine testing described in the next section (Section 2.4.).

### 2.4. MPAS assay testing of ticks submitted in 2023

We tested host-seeking *I. scapularis* ticks that were submitted to CDC for tick pathogen testing in 2023 using a previously described MPAS assay (Hojgaard et al., 2020) and a new MPAS assay that contains the same PCR target for tick-borne pathogens (*Borrelia* spp.: *flaB, Babesia* spp.: *18S, Anaplasma* spp. and *Ehrlichia* spp.: *groEL*) as previously described (Hojgaard et al., 2020; Hahn, 2023) and an additional PCR target (tick 16S mt-rRNA) for tick identification as described in Osikowicz et al., 2023a and Osikowicz et al.,2024. The detection and differentiation of *A. phagocytophilum* variants is based on DNA sequencing of the *groEL* target.

The PCR reactions for these MPAS assays were performed in 25 μl, which included 12.5 μl TEMPase 2x master mix (AMPLICON, Denmark), 10 μl tick nucleic acids extract and 2.5 μl PCR primers resuspended in PCR grade water (see Table 1 for primers and primer concentrations). The MPAS NGS library preparation and sequencing was performed as described above in Section 2.2.

### 2.5. MPAS sequence analysis

The MPAS NGS sequencing analysis was performed using a publicly available bioinformatics pipeline (GitHub - CDCgov/tick_surveillance) that was designed to process FASTQ files produced from the MPAS assay (Osikowicz et al., 2023b). The input files for the pipeline were modified to include the *Anaplasma* spp. primers and reference sequences used in this study (Supplement A). In brief, this Nextflow pipeline trims adapters and primers using cutadapt v3.5. Trimmed sequences are error-corrected and consolidated into amplicon sequence variants (ASVs) using dada2 v1.22.0. ASVs are aligned to expected target sequences using BLASTN v2.12.0. ASVs that are sufficiently close to expected reference sequences contribute to positive calls for the corresponding pathogen or host target. The expected fragment size for *groEL* and *sdhC* are 359 bp and 254 bp, respectively. Initially, cutadapt trims any reads shorter than 100 bases. Subsequently, dada2 amplicon sequence variants are aligned to expected target reference sequences, and only BLASTN alignments that cover ≥99 % of the query sequence with >90 % 95 % identity are considered positive for the corresponding target. In other words, BLASTN alignments must completely cover the expected target sequence and partial alignments will not be considered positive. The minimum percent query aligned (typically 99 %) and minimum percent alignment identity (typically 90 % 95 %) are configurable for each target sequence (Altschul et al., 1990; Callahan et al., 2016; Di Tommaso et al., 2017). In our analysis the ASVs were aligned to the input reference sequences using a 90 % minimum sequence identity parameter. This was done, despite all our ASVs being >99 % identical to the reference sequences, in order to capture potential sequences that are fairly dissimilar to the target sequences. A sample was considered positive for *A. phagocytophilum* if it contained a minimum of 50 reads that aligned to the input reference sequences as previously described in Hojgaard et al., 2020.

### 2.6. Defining pathogen presence by variant and county

For the purposes of this study, counties were classified as “pathogen detected” for *A. phagocytophilum* if at least one *I. scapularis* nymph or adult tested positive for undifferentiated *A. phagocytophilum*, Ap-ha, or Ap-non ha. That is, the Ap “any variant” category includes ticks that tested positive for *A. phagocytophilum* (strain not differentiated) by TaqMan PCR that were not further tested using NGS, as well as aggregated results for Ap-ha and Ap-non ha variants. Counties were classified as “pathogen detected” for each individual variant (Ap-ha, Ap-non ha) if at least one *I. scapularis* nymph or adult tested positive for the variant using NGS. Counties were classified as “not detected” if no nymphal or adult *I. scapularis* tested positive.

### 2.7. Estimating pathogen prevalence by variant and tick life stage by state

We estimated the prevalence of *A. phagocytophilum* variants (undifferentiated, Ap-ha, Ap-non ha) in host-seeking *I. scapularis* nymphs and adults per state in the eastern US using two methods. For ticks tested by TaqMan PCR (Section 2.1), we report the percentage of nymphs or adults tested per state that were positive for *A. phagocytophilum* (strain not differentiated). For states where infected ticks were retested using the duplex MPAS assay (Section 2.2) to differentiate variants, we estimated the proportion of ticks infected with Ap-ha, then estimated the prevalence of Ap-ha per state by scaling the total number of infected ticks (based on TaqMan testing) by the proportion that were infected with Ap-ha. For ticks tested in 2023 that were tested using the MPAS assay, we report the percentage of nymphs or adults infected with either variant (number infected per number tested).

### 2.8. Mapping

County presence tables for *A. phagocytophilum* variants (any variant, Ap-ha, and Ap-non ha) were joined to a US census county-level geographic information system (GIS) layer using Federal Information Processing Standards (FIPS) codes, and data layers were incorporated into maps using ArcMap (version 8.2, ESRI, Redlands, CA). Additionally, GIS layers containing *I. scapularis* county presence and the estimated range of suitable habitat for the tick (Eisen et al., 2016; Hahn et al., 2016; M.B. 2017; CDC, 2023a) were added to contrast the range of reported *I. scapularis* presence to counties where ticks were tested in this study and where *A. phagocytophilum* variant presence and/or prevalence are reported in this analysis.

## 3. Results

### 3.1. Differentiating A. phagocytophilum variants in I. scapularis ticks previously identified as positive by TaqMan

To support ongoing research and surveillance activities, CDC tested 15,757 *I. scapularis* nymphs and 14,103 adults using a previously published TaqMan testing algorithm (Graham et al., 2018). A total of 324 nymphs (2.1 %) and 487 adults (3.4 %) yielded inconclusive results, usually because DNA was scored as poor quality. Of ticks yielding conclusive results, 549 nymphs (3.6 % of 15,433) and 761 adults (5.6 % of 13,616) were infected with *A. phagocytophilum*.

Among these *A. phagocytophilum*-infected ticks, 351 nymphs and 456 adults were retested to differentiate variants. Of the 351 nymphs that were retested, 50 % (*n* = 176) were infected with Ap-ha and 50 % (*n* = 175) were infected with Ap-non-ha. Of the 456 adults that were retested, 373 (81.8 %) were infected with Ap-ha and 83 (18.2 %) were infected with Ap-non-ha (Tables 2–3). No ticks were coinfected with both variants (Tables 2–3).

### 3.2. Amplicon sequences obtained from the two loci sdhC and groEL

We observed all the same Ap-ha and Ap-non-ha sequences for both *sdhC* and *groEL* as reported from a previous study (Hojgaard et al., 2022), with >99.2 % identity to the reference sequences that were also originally reported in Hojgaard et al., 2022 (Supplemental A). New to this study was a sequence for Ap-ha *sdhC* that, as the original reference sequence, is 254 bp but differ at 2 bp and are 99.2% identical. A total of 41 tick samples were positive for this new Ap-ha variant (*sdhC*_Ap-ha-2) and the Ap-ha variant *groEl* sequence *groEl*_Ap-ha-1. All sequences were submitted to GenBank (PP751631-PP751639)

### 3.3. Comparison of variant classification based on sdhC vs groEL

Of the 807 tick DNA samples that yielded a usable DNA amplicon sequence for both the *groEL* and *sdhC* target, there was 100 % concordance between the sequenced targets in determining if the *A. phagocytophilum* variant was categorized as Ap-ha or Ap-non ha.

### 3.4. Detecting and differentiating A. phagocytophilum variants in I. scapularis tested in 2023

Beginning in 2023, all ticks received by CDC were tested using an NGS assay that detects and differentiates *A. phagocytophilum* variants based on *groEL* DNA sequences (Hojgaard et al., 2020; Osikowicz et al., 2024). Among 726 *I. scapularis* nymphs tested, 20 yielded inconclusive result, due to poor quality sample DNA. Among the remaining 706 nymphs tested, 15 (2.1 %) were infected with Ap-ha and seven (1.0 %) were infected with Ap-non-ha. Of 2961 adults tested, 47 yielded inconclusive results due to poor quality sample DNA. Of the 2914 *I. scapularis* adults yielding conclusive sequences, 2.8 % (*n* = 82) were infected with Ap-ha and 1.4 % (*n* = 43) were infected with Ap-non-ha (Table 4). Again, not a single tick was coinfected with both variants.

### 3.5. Geographic distribution of AP variants

A previous study demonstrated equivalent sensitivity in detection of *A. phagocytophilum* between the TaqMan and NGS assays (Hojgaard et al., 2020). In the section above, we confirmed that sequencing the *groEL* target yields variant classifications identical to *sdhC*. We used those findings to justify combining the testing results from the TaqMan (Graham et al., 2018), duplex NGS *A. phagocytophilum* assay (this study), and the multi-pathogen NGS assays (Hojgaard et al., 2020; Osikowicz et al.,2024) to present maps of the county level distribution of *A. phagocytophilum* any variant, Ap-ha and Ap-non ha variants.

**Fig. 1.**
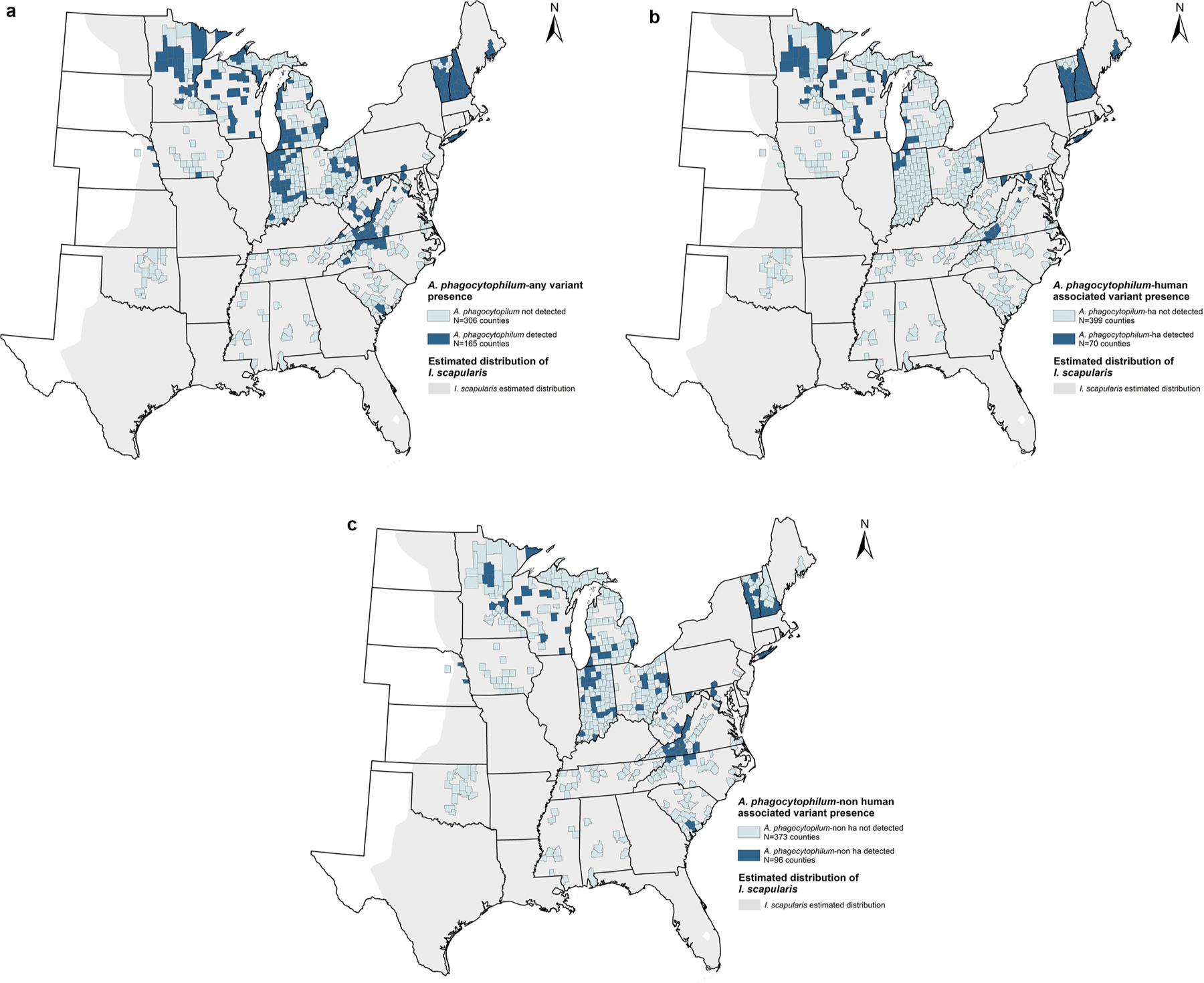
a-c. County-level distribution of *Anaplasma phagocytophilum* in host-seeking *Ixodes scapularis* nymphs and adults submitted to the Centers for Disease Control and Prevention (CDC). Counties shaded dark blue are those where at least one *I. scapularis* nymph or adult tested positive for (a) *A. phagocytophilum*-any variant, (b) *A. phagocytophilum*-human associated (ha) variant, and (c) *A. phagocytophilum*-non human associated (non ha) variant, relative to the estimated distribution (light gray shading) of *I. scapularis* (Hahn et al., 2016, 2017). Counties where ticks were tested by CDC and no *A. phagocytophilum* was detected are shaded light blue.

**Fig. 2.**
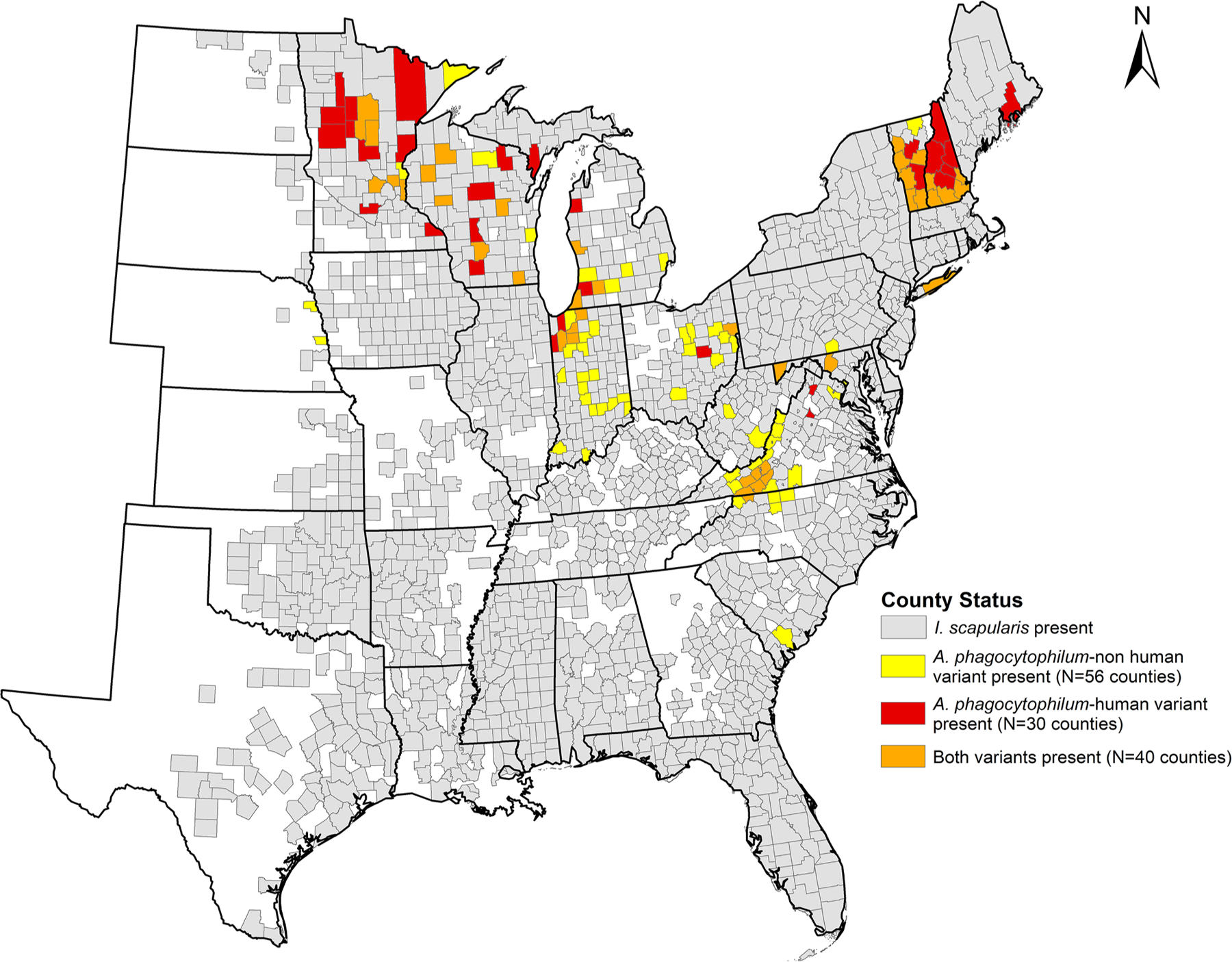
County-level distribution of *Anaplasma phagocytophilum*-non human associated variant (yellow), human associated variant (red), and both variants (orange) in host-seeking *Ixodes scapularis* nymphs and adults submitted to the Centers for Disease Control and Prevention, relative to the county-level distribution (gray) of *I. scapularis* (CDC, 2023). Counties where *A. phagocytophilum* variants are shaded present are those where at least one *I. scapularis* nymph or adult tested positive for the respective variant using next generation sequencing (Hojgaard et al., 2020; 2022).

Ticks were tested from 471 counties representing 23 states (Fig. 1). *Anaplasma phagocytophilum* (any variant) was detected in 165 (35.0 %) counties in 18 states situated in the northeastern, upper midwestern, and southeastern US (Fig. 1a). Ap-ha had a more limited distribution, identified in 70 (14.9 %) of the 469 counties from which ticks were tested spanning 12 of the 23 states (Fig. 1b). Ap-non ha was detected in 96 (20.4 %) of counties spanning 15 of the 23 states from which ticks were tested (Fig. 1c). Ap-ha was the only variant detected in 30 counties. Ap-non ha was the only variant detected in 56 counties. Both variants were detected in 40 counties (Fig. 2).

### 3.6. Prevalence of A. phagocytophilum variants in I. scapularis by state

Prevalence of *A. phagocytophilum* (not differentiated) was estimated in 21 states and the District of Columbia (Washington, D.C.) using TaqMan PCR. As expected, prevalence of *A. phagocytophilum* trended higher in adults compared with nymphs (Tables 2–3). Among states, prevalence of *A. phagocytophilum* (not differentiated) ranged from 0 % to 19 % in adults (Table 2) and from 0 % to 7.8 % in nymphs (Table 3). Among infections that were differentiated by variant, the proportion categorized as Ap-ha differed widely among states (Tables 2–3). We estimated that prevalence of Ap-ha variant ranged among states from 0 % to 16 % in adults (Table 2), and from 0 % to 4.6 % in nymphs (Table 3).

We tested nymphs and adults submitted in 2023 from 11 states using the MPAS assay that can detect and differentiate variants. Across these 11 states, prevalence of Ap-ha ranged from 0 % to 8.9 % in adults and 0 % to 5.1 % in nymphs. Prevalence of Ap-non ha ranged from 0 % to 6 % in adults and 0 % to 6 % in nymphs (Table 4).

## 4. Discussion

Consistent with previous studies conducted at smaller spatial scales (Massung et al., 2002; Courtney et al., 2003; Lee et al., 2014; Keesing et al., 2014; Prusinski et al., 2023), we show that Ap-ha and Ap-non ha are not evenly distributed across the landscape and grouping the variants into aggregated or undifferentiated *A. phagocytophilum* presence or prevalence metrics may inflate anaplasmosis risk. Although we identified significant overlap in the ranges of the variants, with both variants co-occurring in nearly a third (32 %) of counties where either variant was detected, the geographic distribution of Ap-ha was narrower than the distribution of Ap-non ha. Ap-ha was detected primarily in the upper Midwest, Ohio Valley, and Northeast, with a notable cluster in southern Virginia. Ap-non ha was detected more broadly within those regions and also detected in states extending further south and west of the Ap-ha foci. Ap-ha was detected more commonly than Ap-non ha in host-seeking nymphs and adults within states reporting high incidence of anaplasmosis from 2015 through 2019: Minnesota, Wisconsin, New York, Vermont, New Hampshire (CDC, 2023b).

By performing the sequence idnetification analysis with a 90 % sequence homology to the reference sequences, we broaden the chances of discovering new sequences for the two loci (*sdhC* and *groEL*). We did observe a new sequence for Ap-ha *sdhC* (*sdhC*_Ap-ha-2) that is 99.2% identical to the previously described sequence for Ap-ha *sdhC* (*sdhC*_Ap-ha-1). A total of 41 tick samples were positive for this new Ap-ha variant (*sdhC*_Ap-ha-2) and the Ap-ha variant *groEl* sequence *groEl*_Ap-ha-1. Future studies may provide answers to if both Ap-ha *sdhC* variants are found in humans and if they present with different or similar clinical manifestations.

Our study was limited to states that submitted ticks to CDC for testing. As a result, there are substantial gaps in the maps from states that conduct their own tick testing or are not engaged in tick surveillance activities. For example, despite efforts to characterize the distribution of *A. phagocytophilum* variants in Pennsylvania and New York, very few counties from those states were represented in our study. Over two decades ago, Courtney et al. (2003) showed that prevalence of *A. phagocytophilum* in host-seeking *I. scapularis* adults was significantly lower in northwestern sites compared with southeastern sites in Pennsylvania, but in the northwest, only the Ap-ha variant was detected; both variants were detected in the southeast. While that study showed geographic differences in the representation of the two variants, the numbers of host-seeking ticks tested per location were extremely limited making it difficult to generalize these findings. In New York, tick surveillance efforts are robust and have been on-going for nearly two decades. Based on genotyping of host-seeking *A. phagocytophilum*-infected *I. scapularis* adults (3207) and nymphs (1183) collected across New York from 2008 to 2020, Ap-ha was more prevalent (5.4 %) in adults compared with Ap-non ha (categorized as variant 1 in that study; 1.7 %) (Prusinski et al., 2023). By contrast, nymphs had a higher prevalence of Ap-non ha (2.6 %) compared with Ap-ha (1.7 %). Notably, the relative proportions of genotypes varied over space and time. The study documented expansion of the Ap-ha variant out of the Hudson Valley and Capital District regions and showed that the distribution of Ap-non ha was largely unchanged and was the predominant variant detected in host-seeking ticks in western New York.

In contrast to the study from New York, where collection efforts were reasonably consistent over time with at least a single site visited per county twice per year throughout the 18 years of surveillance (Prusinski et al., 2023), the data collection in our study was not systematic. Ticks were tested from only a fraction of eastern states and the proportion of counties sampled within each state was highly variable. This uneven sampling effort and pooling of ticks over time precluded in-depth analysis of spatio-temporal changes in the distribution or prevalence of *A. phagocytophilum* variants. Nonetheless, by testing host-seeking *I. scapularis* collected across 23 states in the eastern US, we were able to show that Ap-ha has a slightly more limited distribution than Ap-non ha, underscoring the importance of differentiating variants for risk assessment for anaplasmosis. The relative proportions of each variant are likely to vary across very small spatial scales and over time (Keesing et al. 2014).

Previous studies have shown that although the variants share a common vector, *I. scapularis*, they appear to be maintained in independent enzootic cycles with Ap-non ha being common in deer and Ap-ha being common in rodents (Telford et al., 1996; Massung et al., 2003a, b; Massung et al., 2005; Keesing et al., 2014). Owing to spatial and temporal variation in host composition, within geographic regions where both variants co-occur, differences in host-feeding will likely result in changing proportions of ticks carrying Ap-ha relative to Ap-non ha across years and among locations (Keesing et al., 2014). Ap-ha and Ap-non ha are rarely observed together in individual ticks, including in this study, suggesting competitive interaction within the vector (Krakowetz, et al. 2014; Prusinski et al. 2023). Changes in host composition and competition between variants is likely to result in changes in the distribution of risk for anaplasmosis (Keesing et al., 2014; Prusinski et al., 2023).

Related, in part, to the challenges posed by earlier detection methods to differentiate *A. phagocytophilum* variants (Tokarz et al., 2009; R. 2017; Wroblewski et al., 2017; Graham et al., 2018) variant-specific records were lacking across broad regions of the US. Due to the limited number of studies that differentiated variants, a recent compilation of county level records of *A. phagocytophilum* presented the data in aggregate (Fleshman et al., 2022). Through retesting *A. phagocytophilum* infected ticks collected from 2012 through 2022 with an NGS assay designed to differentiate variants and testing more recently collected ticks with a newly described NGS assay, we were able to provide more accurate maps of the risk of encountering human-disease causing variant (Ap-ha) of *A. phagocytophilum*. Notably, Ap-ha distribution maps are incomplete across the range of *I. scapularis* in the eastern US and non-existent across the range of *I. pacificus* in the western US. While we were able to improve upon existing maps, there is a high degree of uncertainty in localities where very few ticks were submitted for testing. Differentiation of variants is necessary to improve accuracy in assessment of human risk for anaplasmosis. Risk is expected to change over time, highlighting the need for continued surveillance and testing of ticks using variant specific assays, such as the assays described here.

## Supplementary Material

supplemental

## Figures and Tables

**Table 1:** PCR primers used in this study.

Organism	PCR Target	Sequence	Concentration (nM)	Reference
*Anaplasma* spp.	*sdhC*	TCGTCGGCAGCGTCAGATGTGTATAAGAGACAGAGTGTCTATAAGCTGCCGATAA GTCTCGTGGGCTCGGAGATGTGTATAAGAGACAGAACATCAACCAACCACTGAA	300	Hojgaard et al., 2022
*Anaplasma/Ehrlichia spp*.	*groEL*	TCGTCGGCAGCGTCAGATGTGTATAAGAGACAGTACTCAGAGTGCTTCTCAATGT GTCTCGTGGGCTCGGAGATGTGTATAAGAGACAGGCATACCATCAGTTTTTTCAAC	300	Hojgaard et al., 2020
*Babesia* spp.	*18S*	TCGTCGGCAGCGTCAGATGTGTATAAGAGACAGGTAATTCCAGCTCCAATAGCGTA GTCTCGTGGGCTCGGAGATGTGTATAAGAGACAGTCTAAGAATTTCACCTCTGACAGT	300	Hojgaard et al., 2020
*Borrelia* spp.	*flaB*	TCGTCGGCAGCGTCAGATGTGTATAAGAGACAGGAGCTTGGAATGCARCCTGC GTCTCGTGGGCTCGGAGATGTGTATAAGAGACAGTCAAGTCTATTTTGRAAAGCAC	300	Hojgaard et al., 2020
*Ixodes scapularis*	Actin	TCGTCGGCAGCGTCAGATGTGTATAAGAGACAGGCCATGTACGTGGCCATCCA GTCTCGTGGGCTCGGAGATGTGTATAAGAGACAGGCTCGGTGAGGATCTTCAT	150	Hojgaard et al., 2020
*Ixodes* spp.	Tick *16S* mt-rRNA	TCGTCGGCAGCGTCAGATGTGTATAAGAGACAGCTGCTCAATGATTTTTTAAATTGCTGTGG GTCTCGTGGGCTCGGAGATGTGTATAAGAGACAGAATTCWTAGGGTCTTCTTGT	150	Osikowicz et al., 2023a

**Table 2:** Proportions of host-seeking *I. scapularis* adults infected with *A. phagocytophilum* by state. Ticks were tested by TaqMan PCR to identify *A. phagocytophilum*; infected ticks were retested with a duplex MPAS to differentiate variants. Confidence in prevalence estimates for sample sizes less than 25 is very low.

	TaqMan testing		Duplex MPAS testing				
State	No. positive/No. tested	Prevalence of Ap-not differentiated (%)	No. retested	No. Ap-ha	No. Ap-non ha	Proportion Ap-ha	Estimated prevalence of Ap-ha (%)
Alabama	0/60	0	0				0
Washington, D. C.	0/3	0	0				0
Iowa	1/60	2	0				n/a*
Indiana	78/3448	2.3	43	21	22	0.49	1.1
Maryland	17/304	5.6	15	11	4	0.73	4.1
Michigan	68/1578	4.3	19	14	5	0.74	3.2
Minnesota	11/201	5.5	9	7	2	0.78	4.3
Mississippi	0/74	0	0				0
North Carolina	37/455	8.1	9	0	9	0	0.0
Nebraska	0/10	0	0				0
New York	6/32	19	6	5	1	0.83	16
Ohio	22/948	2.3	18	1	17	0.06	0.1
Oklahoma	0/75	0	0				0
Pennsylvania	0/12	0	0				0
South Carolina	0/256	0.0	0				0.0
Tennessee	0/213	0.0	0				0.0
Virginia	42/517	8.1	18	7	11	0.39	3.2
Vermont	467/5208	9.0	310	299	11	0.96	8.7
Wisconsin	12/162	7.4	9	8	1	0.89	6.6

* Prevalence was a non-zero value based on TaqMan testing, but no ticks were available for retesting to differentiate strains using MPAS, therefore estimated prevalence of Ap-ha is given as not applicable (n/a).

**Table 3:** Proportions of host-seeking *I. scapularis* nymphs infected with *A. phagocytophilum* by state. Ticks were tested by TaqMan PCR to identify *A. phagocytophilum*; infected ticks were retested with a duplex MPAS to differentiate variants. Confidence in prevalence estimates for sample sizes less than 25 is very low.

State	TaqMan testing			Duplex MPAS testing			
	No. positive	Prevalence of Ap-not differentiated (%)	No. retested	No. Ap-ha	No. Ap-non ha	Proportion Ap-ha	Estimated prevalence of Ap-ha (%)
Alaska	0/5	0	0				0
Washington, D. C.	1/453	0.2	1	0	1	0.00	0.0
Iowa	0/13	0	0				0
Indiana	22/2054	1.1	19	5	14	0.26	0.3
Kentucky	0/13	0	0				0
Maryland	17/708	2.4	12	6	6	0.50	1.2
Maine	5/154	3.3	4	4	0	1.00	3.3
Michigan	55/2037	2.7	8	1	7	0.13	0.3
Minnesota	111/2020	5.5	95	80	15	0.84	4.6
North Carolina	26/1124	2.3	3	2	1	0.67	1.5
New York	48/615	7.8	40	18	22	0.45	3.5
Ohio	0/3	0	0				0
Pennsylvania	3/270	1.1	3	0	3	0.00	0.0
South Carolina	0/5	0	0				0
Tennessee	0/26	0	0				0
Virginia	161/3604	4.5	89	14	75	0.16	0.7
Vermont	48/912	5.3	38	30	8	0.79	4.2
Wisconsin	40/1018	3.9	38	16	22	0.42	1.7
West Virginia	12/399	3.0	1	0	1	0.00	0

**Table 4:** Proportions of host-seeking *I. scapularis* nymphs and adults infected with *A. phagocytophilum* by state. Ticks were tested by MPAS to identify and differentiate *A. phagocytophilum* variants. Confidence in prevalence estimates for sample sizes less than 25 is very low.

State	Life stage	No. tested	No. Ap-ha positive	No. Ap-non ha positive	Proportion Ap-ha	Prevalence of Ap-ha (%)	Prevalence of Ap-non ha (%)
Indiana	Adult	913	5	11	0.31	0.5	1.2
	Nymph	1	0	0		0	0
Maryland	Adult	250	22	14	0.61	8.8	5.6
	Nymph	53	0	0		0	0
Michigan	Adult	106	0	2	0.00	0.0	1.9
	Nymph	35	0	2	0	0	6
Minnesota	Adult	18	0	1	0	0	6
	Nymph	1	0	0		0	0
North Carolina	Adult	43	0	0		0	0
	Nymph	29	0	0		0	0
Nebraska	Adult	78	0	3	0.00	0	4
	Nymph	4	0	0		0	0
New Hampshire	Adult	966	42	8	0.84	4.4	1.0
	Nymph	213	6	2	0.75	2.8	1.0
Ohio	Adult	344	1	2	0.33	0.3	0.6
	Nymph	49	1	1	0.50	2	2
South Carolina	Adult	61	0	1	0.00	0	2
Vermont	Adult	135	12	1	0.92	8. 9	0.7
	Nymph	157	8	0	1.00	5.1	0.0
West Virginia	Nymph	164	0	2	0.00	0.0	1.2

## Data Availability

Data will be made available on request.
